# Oral primary care: an analysis of its impact on the incidence and mortality rates of oral cancer

**DOI:** 10.1186/s12885-017-3700-z

**Published:** 2017-10-30

**Authors:** Thiago Augusto Hernandes Rocha, Erika Bárbara Abreu Fonseca Thomaz, Núbia Cristina da Silva, Rejane Christine de Sousa Queiroz, Marta Rovery de Souza, Allan Claudius Queiroz Barbosa, Elaine Thumé, João Victor Muniz Rocha, Viviane Alvares, Dante Grapiuna de Almeida, João Ricardo Nickenig Vissoci, Catherine Ann Staton, Luiz Augusto Facchini

**Affiliations:** 1 Federal University of Minas Gerais, School of Economics, Center of post-graduate and Research in Administration, Belo Horizonte, Minas Gerais Brazil; 2 Department of Public Health, Federal University of Maranhão, São Luís, Maranhão Brazil; 3 National School of Public Health, Nova University of Lisbon, Lisboa, Portugal; 4 Department of Public Health, Federal University of Goiás, Goiânia, Goiás, Brazil; 5 Faculty of Economics, Department of Administrative Sciences, Federal University of Minas Gerais, Belo Horizonte, Minas Gerais Brazil; 6 Faculty of Nursing, Department of Collective Health, Federal University of Pelotas, Pelotas, Rio Grande do Sul Brazil; 7 Medomai Information Technology Systems, Belo Horizonte, Minas Gerais Brazil; 8 Duke Division of Emergency Medicine, Duke University Health System, Duke Global Health Institute, Duke University, Durham, USA; 9 Duke Division of Emergency Medicine, Duke University Health System, Duke Global Health Institute, Duke University, Durham, USA; 10 Faculty of Medicine, Department of Social Medicine, Federal University of Pelotas, Pelotas, Rio Grande do Sul Brazil; 11 Business Administration Department – Observatory of human resources for health, Universidade Federal de Minas Gerais, Antonio Carlos, avenue, 6627, Belo Horizonte, Minas Gerais Brazil

**Keywords:** Health systems, Health inequalities, Mortality, Mouth neoplasms, Ecological studies, Primary health care, Program evaluation

## Abstract

**Background:**

Oral cancer is a potentially fatal disease, especially when diagnosed in advanced stages. In Brazil, the primary health care (PHC) system is responsible for promoting oral health in order to prevent oral diseases. However, there is insufficient evidence to assess whether actions of the PHC system have some effect on the morbidity and mortality from oral cancer. The purpose of this study was to analyze the effect of PHC structure and work processes on the incidence and mortality rates of oral cancer after adjusting for contextual variables.

**Methods:**

An ecological, longitudinal and analytical study was carried out. Data were obtained from different secondary data sources, including three surveys that were nationally representative of Brazilian PHC and carried out over the course of 10 years (2002–2012). Data were aggregated at the state level at different times. Oral cancer incidence and mortality rates, standardized by age and gender, served as the dependent variables. Covariables (sociodemographic, structure of basic health units, and work process in oral health) were entered in the regression models using a hierarchical approach based on a theoretical model. Analysis of mixed effects with random intercept model was also conducted (alpha = 5%).

**Results:**

The oral cancer incidence rate was positively association with the proportion of of adults over 60 years (β = 0.59; *p* = 0.010) and adult smokers (β = 0.29; p = 0.010). The oral cancer related mortality rate was positively associated with the proportion of of adults over 60 years (β = 0.24; *p* < 0.001) and the performance of preventative and diagnostic actions for oral cancer (β = 0.02; *p* = 0.002). Mortality was inversely associated with the coverage of primary care teams (β = −0.01; *p* < 0.006) and PHC financing (β = −0.52^−9^; *p* = 0.014).

**Conclusions:**

In Brazil, the PHC structure and work processes have been shown to help reduce the mortality rate of oral cancer, but not the incidence rate of the disease. We recommend expanding investments in PHC in order to prevent oral cancer related deaths.

**Electronic supplementary material:**

The online version of this article (10.1186/s12885-017-3700-z) contains supplementary material, which is available to authorized users.

## Background

Head and neck cancers are currently the seventh most common malignancy worldwide, with more than 600,000 new cases diagnosed each year; oral cancer is responsible for approximately half of these cases [1]. The incidence of oral cancer is increasing; furthermore, it is not evenly distributed globally [2]. While India and France have the highest incidence rates by country, South America has the highest incidence rates compared to other continents. Brazil in particular has a rising incidence rate, [3, 4] with a projection of 16,340 new cases in 2016 [5]. Its distribution is heterogeneous among Brazilian cities, with approximately 30% of cases occurring in capital cities [4]. The oral cancer incidence is also higher in men and increases with age [5, 6].

The etiology of oral cancer is multifactorial including endogenous (genetic predisposition) and exogenous (environmental and behavioral) factors [7–10]; smoking and alcohol consumption are the largest risk factors [7–11]. Depending on the type and stage of diagnosis, oral cancer can be managed, treated, and cured [12]. Yet studies addressing the role of primary health care (PHC) in the control and reduction of oral cancer and its sequelae are scarce [13]; similarly, there is limited evidence on the impact of public health prevention initiatives on oral cancer incidence and mortality [14].

In Brazil, PHC is the preferred entry into the public health system (Universal Health System – SUS) and can serve as a place to identify risk factors, perform early diagnostics, and provide basic care for cancer patients [13, 15]. Beginning in 2004, the National Oral Health Policy included the diagnosis of oral cavity lesions in the scope of PHC examinations [16, 17]. Primary care professionals should perform oral examinations routinely, enabling the detection of early stage cancers [18–21] and increasing the chances of cure and survival [12]. However, despite advances in expanding access to dental services, there are still major challenges in the structure and work process of PHC [22–25]. Currently, there is a low level of inclusion of dental practitioners in early detection initiatives [21]; furthermore, in 2016 the PHC oral health policy covered only 37% of the Brazilian population [26, 27]. Problems cited throughout the Brazilian PHC system include a lack of preventive screening actions [13, 28], gaps in professional training [21, 28] and socioeconomic inequities [29–31].

Establishing a diagnostic network that allows primary care services to identify potentially malignant lesions is an important step in reducing the number of individuals first seeking medical care at an advanced stage of the disease [29, 32, 33]. The proportion of patients diagnosed at advanced stages of the disease has not changed in the last 40 years [32, 34]. Evidence indicates that well structured PHC could reduce the incidence and mortality due to oral cancers [33–36]. However, the role of the structure and work process of oral primary care, namely coverage, supply availability, and prevention activities, is still not well-defined in low and middle income countries.

Considering the evidence discussed so far and the lack of long-term and population-based studies, the aim of this study was to analyze the effect of the parameters related to the PHC structure and work process on the incidence and mortality rates of oral cancer. It was hypothesized that better coverage, supply availability, and prevention activities in primary public care services will have a positive impact on reducing incidence and mortality due to oral cancer in Brazil.

## Methods

### Study design and area

This is an ecological, longitudinal, and analytical study. The unit of analysis was comprised of the Brazilian Federative Units (BFU). Brazil has 5570 municipalities distributed in 27 states (BFU = 27), divided into five geopolitical regions (North, Northeast, Southeast, South and Midwest). Only previously collected data was used in this study, and no participants were involved.

### Data sources

We compiled data from eleven different data sources with the Brazilian Health System records, census data, and measures of socioeconomic development. Data was categorized as indicators of either sociodemographic, structure, work process and results aspects (additional file 1). All these databases are publically accessible.

Since we were conducting a multi-sourced secondary data analysis, we chose to aggregate the data at the Brazilian Federal Unit level and included data from a 10 year time span. This is the best strategy for rare outcomes, and linking the datasets by BFU allowed for better data quality and availability.

#### Surveys databases

Between 2001 and 2002, family health strategy teams (FHST) were implemented in all Brazilian states, leading to the first primary care monitoring censusAll BFU with FHST registered in the PHC information system as of May 2001 were included in this study. Data was collected from June 2001 to August 2002.

In 2008 a sampling survey was conducted; variables on organizational dynamics and labor were included and aspects of the 2001–2002 study were kept to ensure comparability across studies. Brazilian municipalities with FHST were stratified based on population size and Human Development Index (HDI) dimension scores. Data was collected between June 2008 and November 2008 by the Observatory of Human Resources in Health, from School of Economics of the Federal University of Minas Gerais.

For both surveys, the primary respondent was a nurse, or a general practitioner if a nurse was unavailable. This was because of the nature of the data collected and to ensure the legitimacy of the data collected. In the case of the oral health instrument, the primary respondent was the dentist.

The third survey was part of the National Program for Improving Access and Quality of Primary Care (PMAQ-AB) [37]. The data collected was similar to the two prior surveys, allowing for comparison. Basic health units (BHU) located in prisons, schools, mobile units, or boats were not included. The evaluation of the work process included only data of nearly half BHU existing in Brazil. In the first PMAQ-AB cycle, the Ministry of Health set a maximum adherence rate of no more than 50% of primary care teams per municipality. However, for the physical structure characterization, all BHU of Brazil were visited. The collection of PMAQ-AB data was carried out between May 2012 and October 2012.

#### Administrative databases

Primary Care Information System (SIAB) [27] is dedicated to monitoring actions and outcomes of Brazilian primary care programs. SIAB is composed of data on family registries, health coverage, living conditions, health status, and health team composition. We used this database to collect information on the number of PHC and oral health teams (OHT), as well as preventive activities performed for the purpose of detecting oral cancer.

System for Specialized Management Support (SAGE) is a business intelligence panel designed to provide information to support decision-making, management, and knowledge generation in healthcare [26]. This system is responsible for providing financial data invested in PHC.

Ambulatory Information System (SIA-SUS) was conceived in 1992 and is the system responsible for summarizing all out-patient procedures performed by public health services [27]. There is a large volume of available data, including data regarding oral health procedures performed by primary care teams, which were considered in this study.

#### Sociodemographic databases

United Nations Development Programme (UNDP) is a United Nation programme working in nearly 170 countries and territories with the goal of eradicating poverty and reducing inequalities and exclusion [38]. We obtained the HDI index from UNDP databases.

Brazilian Institute of Geography and Statistics (IBGE) [39] is an institution that publishes data on Brazilian economic activities, population projections, and geoscience. Quantitative information regarding the population and Gini index were extracted from IBGE databases. Population size was used to compute the adjusted proportional rates.

#### Epidemiological databases

The Mortality Information System (SIM) was created by the Brazilian Ministry of Health in 1975. The system summarizes information on mortality in every Brazilian municipality and is updated monthly. We collected data on mortality due to oral cancer from this system. [27]. For analytical purposes, we considered oral cancer all ICD codes comprised between C00 and C10.

Surveillance of both risk and protective factors for chronic diseases through telephone survey (VIGITEL) [26, 40] is a regular research in Brazil. The aims of telephone surveys are to monitor the frequency and distribution of risk and protective factors for non-communicable diseases in all capitals of the 26 Brazilian states and the Federal District. Interviews are conducted by randomly sampling each citiy’s adult population living in households with a landline. Data on the proportion of adult smokers in each city was collected and evaluated by VIGITEL.

The National Cancer Institute (INCA) is an auxiliary institution of the Ministry of Health that develops and coordinates integrated actions for the prevention and control of cancer [5]. INCA databases were used to collect informations about the estimated number of cases of oral cancer per year in Brazil.

### Theoretical model

According to Donabedian [41], structural features may influence the quality of care processes and, as a result, affect a patient’s health status. The three elements of structure, process and outcome may also be controlled by socioeconomic and demographic factors. Additionally, there is a lag effect between care supply and its effects [42]. Therefore, in this study, sociodemographic, structure and work process context data are analyzed over a time span of 10 years, even if outcome indicators are not yet present. Studies on how the different structure, process and outcome elements fit together are scarce despite their relevance. Structure elements, mainly composed of financial variables, human resources and physical infrastructure, and process elements, which reflects the daily practice of care supply, are the important proxies for a deeper understanding of the impact of care provision actions on health outcomes.

**Fig. 1:**
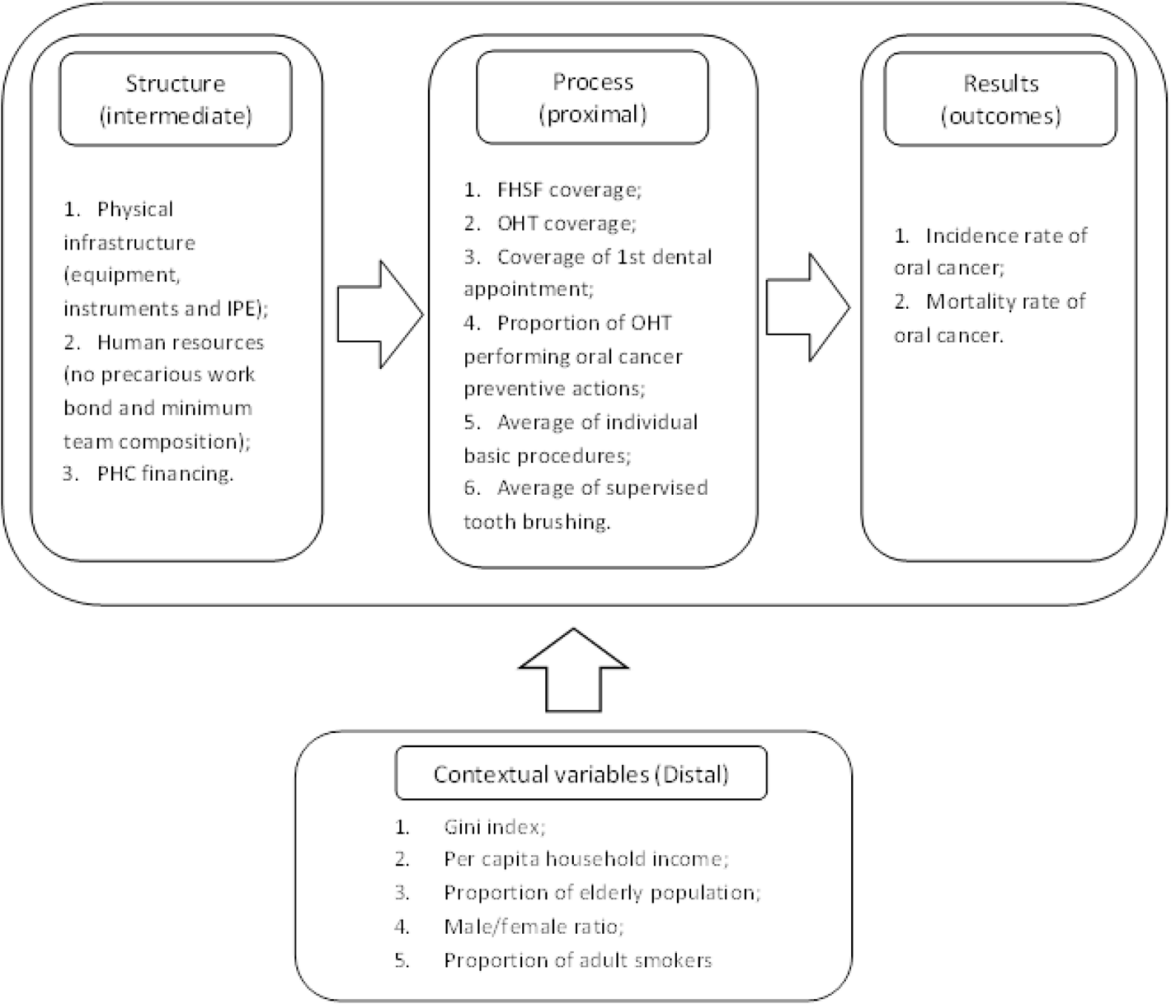
Theoretical model of factors associated with incidence and mortality rates of oral cancer

In the proposed model, FHST and OHT coverage were considered work process indicators, since the Family Health Strategy is a reorientation of the health care model. Therefore, it is assumed that coverage expansion contributes to the consolidation of the new process for health service provision. This theoretical model (Fig. 1) examines the relationship between the structure elements, processes, and outcomes related to oral cavity cancer, as well as the mediating effects of sociodemographic variables.

### Data analysis

Mortality rates were standardized by sex and age using the direct method compared to the Brazilian population as reference. It was not possible to standardize incidence rates since oral cancer is not a mandatory reporting event in Brazil; therefore, the data collected by our sources are not stratified by demographic variables. Descriptive analysis was quantitatively represented by means with standard deviations, percentiles and medians of the study indicators for Brazil.

Since this is a study with a hierarchical structure of longitudinal data, we opted for the analysis of mixed effects with a random intercept model. In this analysis, the coefficient is fixed, but the intercept is random, allowing for the incorporation of the effect of the random intercept in the analytical structure (43,44). This modeling allows analyzing unbalanced longitudinal data (measurements in each BFU observed at different times) in hierarchical structure, incorporating the dependency, variance, and covariance matrix of units [43].

Coefficients of mixed effects (β) and 95% confidence intervals (95%CI) were estimated. We built unadjusted and adjusted models for both outcomes: incidence rates (Model 1) and mortality of oral cancer (Model 2). A hierarchical modelling approach was adopted. Variables were kept for the adjusted model if they had significance of 0.1 at each level. Both models were first adjusted for sociodemographic and contextual variables. Next, the structure indicators of public primary health care services and work process were included. A cutoff of 5% was considered as the criterion for statistical significance (α = 0.05). Multicollinearity among variables of the same block was tested. Analyses were performed using Stata software, version 11.0 (StataCorp., CollegeStation, TX, USA). The construction of maps with the Brazilian geopolitical distribution and the incidence and mortality rates of oral cancer were made with ArcGIS software version 10.2.

## Results

**Fig. 2:**
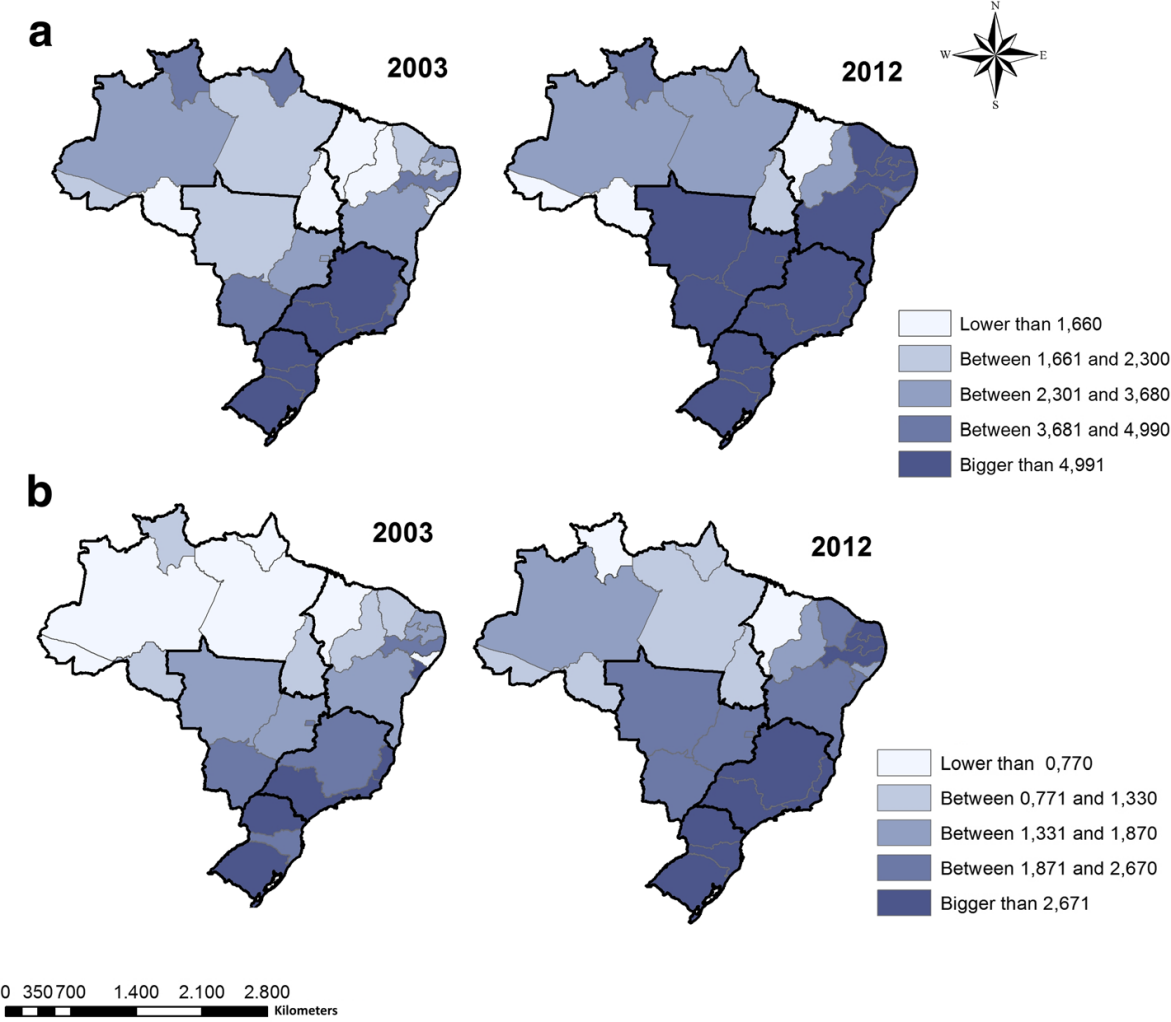
Incidence and mortality rates for oral cancer in Brasil. 2003 and 2012

During the study period the mortality rate adjusted per 100,000 inhabitants varied between 1.70 deaths in 2003 to 2.51 deaths in 2012. The incidence rate fluctuated from 3.62 in 2003 to 5.31 in 2012. While incidence rates did not vary over time, mortality rates increased between 2003 and 2012 (Fig. 2). The socioeconomic and demographic characteristics seen between 2002 and 2012 are presented in Table 1. The percentage of BHU with the minimum equipment for dental office operation varied among evaluated years, with the highest percentages in 2002 (90.9%) and 2012 (95.5%). Instruments for the clinical examination performance and individual protection equipment were part of the structure of 99.2% of BHU in the country in 2008, for example. The percentage of complete healthcare team remained similar between 2002 and 2008, but declined in 2012. The percentage of dentists with a legally protected contractual relationship increased from 30.4% in 2002 to 57.3% in 2008. In the work process, the percentage of preventive measures and diagnosis of oral cancer within the PHC was 49.9% in 2008 and rose to 74.5% in 2012 (Table 2).

**Table 1:** Socio-demographic characteristics of Brazilian municipalities, 2000–2012

Year	Gini Index		Percentage of elderly population		Male/female ratio (M/F)		Proportion of adult smokers		Per capita household income		PHC financing (in millions)		Coverage of Family Health Strategy Teams		Coverage of Oral Health Teams	
	x	sd	x	sd	x	sd	x	sd	x	sd	x	sd	x	sd	x	sd
2002	0.57	0.03	7.42	1.87	98.53	3.91			526.40	211.92	119.89	111.89	41.24	19.85	16.36	12.06
2003	0.56	0.03	7.41	1.87	98.53	3.91			495.81	198.34	136.35	129.84	44.90	20.85	22.32	16.01
2004	0.56	0.04	7.40	1.86	98.52	3.91			507.03	203.54	164.86	151.13	48.63	21.22	31.71	22.01
2005	0.55	0.03	7.38	1.86	98.50	3.92			537.07	218.13	192.49	168.28	55.15	21.71	45.93	30.09
2006	0.54	0.04	7.37	1.86	98.50	3.92			594.12	232.86	224.97	196.20	55.80	21.63	48.92	30.56
2007	0.54	0.04	8.29	2.08	98.35	4.04	15.72	2.61	617.36	253.05	252.80	221.97	56.19	21.09	51.85	30.97
2008	0.53	0.04	8.53	2.14	98.41	4.15	14.73	3.29	649.98	256.73	285.68	248.96	59.22	20.77	54.49	31.17
2009	0.53	0.04	8.74	2.21	98.39	4.23	14.84	3.26	680.47	258.75	335.89	294.11	61.28	20.58	59.65	34.29
2010	0.60	0.04	9.42	2.26	97.57	3.75	14.34	2.97	675.23	281.23	362.10	316.69	62.52	19.93	62.88	32.06
2011	0.52	0.03	9.41	2.26	97.57	3.75	13.36	3.48	722.71	275.77	412.20	363.76	61.54	18.84	63.54	30.94
2012	0.52	0.04	9.40	2.26	97.57	3.76			777.33	272.16	468.21	425.48	62.67	17.81	66.46	29.25

*PHC* Primary health care, *x* Mean, *sd* Standard deviation

**Table 2:** Average suitability of structure elements and work processes related to coping with oral cancer. Brazil, 2002–2008-2012

Year	BRAZIL		
	2002	2008	2012
% Full team (modality I)^a^			
Mean	98.7	98.0	88.1
Standard deviation	8.1	9.7	21.3
Q1	100.0	100.0	100.0
Median	100.0	100.0	100.0
Q3	100.0	100.0	100.0
% Dentist with legally protected work contract PHC			
Mean	30.4	57.3	–
Standard deviation	45.4	46.1	–
Q1	0	0	–
Median	0	100.0	–
Q3	100.0	100.0	–
% BHU with minimum equipment			
Mean	90.9	64.5	95.5
Standard deviation	13.7	12.1	10.2
Q1	88.9	61.5	100.0
Median	100.0	61.5	100.0
Q3	100.0	61.5	100.0
% BHU with instruments (clinical examination)			
Mean	81.0	99.2	–
Standard deviation	39.2	9.0	–
Q1	100.0	100.0	–
Median	100.0	100.0	–
Q3	100.0	100.0	–
% prevention actions/cancer diagnosis			
Mean	–	49.9	74.5
Standard deviation	–	24.0	12.6
Q1	–	35.7	66.7
Median	–	50.0	75.0
Q3	–	71.4	83.3

(−-) not rated. Q1: first quartile. Q3: third quartile. BHU: Basic Health Units. PHC: Primary health care. ^a^Including at least 01 dentist and 01 advanced dental hygiene practitioner (ADHP) or 01 dental hygiene practitioner (DHP)

In the unadjusted analyses, incidence rates of oral cancer were higher in states with a higher per capita household income (β = 0.004, *P* = 0.001), higher proportion of older subjects (β = 0.370, *P* = 0.020), lower gender ratio (β = −0230, *P* < 0.001), higher proportion of adult smokers (β = 0.37, *P* = 0.024), lower FHST coverage (β = −0030, *P* = 0.005), lower mean of supervised tooth brushing (β = −0340, *P* = 0.039), and had municipalities with a higher proportion of FHST performing preventitive oral cancer care (β = 0.008, *P* = 0.014). Positive correlations were also found between mortality rates for oral cancer and per capita household income (β = 0.007, P < 0.001), proportion of elderly subjects (β = 0.190, P < 0.001), and performance of disease control measures (β = 0.020, *P* = 0.002). Negative correlations were found with gender ratio (β = −0.050, P < 0.001) and FHST coverage (β = −0004, *P* = 0.032), as shown in Table 3.

**Table 3:** Unadjusted association between contextual variables, structure, work process and results and incidence and mortality rates of oral cancer in Brazil

Variables	Incidence rate of oral cancer (model 1)					Mortality rate of oral cancer (model 2)				
	Fixed Effect			Random effect		Fixed Effect			Random effect	
	β	CI95%	P	β	Residue	β	CI95%	P	β	Residue
Contextual variables										
Gini index	5.57	−6.63: 17.77	0.371	2.96	1.79	−0.68	−2.11: 0.75	0.354	0.89	0.50
Per capita household income	0.004	0.002: 0.007	0.001	2.35	1.72	0.007	0.0003: 0.001	<0.001	0.81	0.49
Proportion of elderly population	0.37	0.06: 0.68	0.020	2.32	1.77	0.19	0.14: 0.23	<0.001	0.58	0.45
Male/Female ratio	−0.23	−0.35: −0.10	<0.001	2.50	1.71	−0.05	−0.07: −0.03	<0.001	0.79	0.47
Proportion of adult smokers	0.37	0.04: 0.69	0.024	2.59	0.97	−0.006	−0.04: 0.03	0.779	0.93	0.45
Structure of PHC services										
Financing of PHC	−0.24^−9^	−0.19^−9^: 0.14^−9^	0.775	2.92	1.80	−0.27^−9^	−0.55^−9^: 0.15^−10^	0.063	0.94	0.49
% full team (modality 1)	−0.002	−0.11: 0.10	0.965	2.86	1.65	0.01	−0.009: 0.03	0.274	0.87	0.49
% full team (modality 2)	−0.04	−.026: 0.18	0.743	2.85	1.77	−0.004	−0.03: 0.02	0.778	0.84	0.52
% team with no precarious work bond (modality 1)	0.006	−0.02: 0.04	0.658	2.74	1.78	0.004	−0.001: 0.01	0.144	0.80	0.51
% team with no precarious work bond (modality 2)	0.006	−0.02: 0.03	0.682	2.75	1.78	0.004	−0.002: 0.01	0.209	0.81	0.51
% adjustment of oral health equipment	0.14	−0.006: 0.28	0.060	2.58	1.61	0.002	−0.006: 0.009	0.682	0.88	0.49
% adjustment of examination instruments	0.01	−0.04: 0.06	0.622	2.74	1.77	−0.0008	−0.01: 0.01	0.904	0.84	0.52
% adjustment of IPE inputs	−0.02	−0.16: 0.12	0.794	2.86	1.65	0.01	−0.003: 0.02	0.121	0.87	0.48
Work process in PHC										
% of actions for prevention and diagnosis	0.08	0.01: 0.14	0.014	2.51	1.57	0.02	0.009: 0.04	0.002	0.76	0.46
Products of PHC services										
FHST Coverage	−0.03	−0.05: −0.009	0.005	2.65	1.74	−0.004	−0.007: −0.0003	0.032	0.88	0.49
OHT Coverage	−0.006	−0.02: 0.008	0.408	2.80	1.80	0.002	−0.0007: 0.08	0.170	0.92	0.49
Mean supervised tooth brushing	−0.34	−0.66: −0.02	0.039	3.09	1.54	−0.05	−0.10: 0.01	0.112	0.96	0.49
Coverage of 1st dental consultation	–	–	–	–	–	−0.02	−0.06: 0.03	0.502	0.87	0.54
Mean individual basic procedures	−1.49	−4.38: 1.39	0.311	3.05	1.84	−0.06	−0.46: 0.34	0.765	0.89	0.50

*β* regression coefficient, *CI95%* 95% confidence interval, *P* Type I error probability (α). (−-) not rated

In the multivariable analyses, oral cancer incidence rates remained positively associated with a higher proportion of elderly subjects (β = 0.96; P < 0.001) and higher proportion of adult smokers (β = 0.29; *P* = 0.010). Higher mortality rates were recorded in municipalities with higher proportion of elderly subjects (β = 0.24; P = <0.001), higher proportion of control actions for oral cancer (β = 0.02; P = 0.002), lower FHST coverage (β = −0.01, *P* = 0.006), and less public funding for PHC actions (β = − 0.52^−9^; P = 0.014). Table 4 further outlines the results of the multivariable analysis.

**Table 4:** Variables associated with incidence and mortality rates of oral cancer in Brazil (per 100,000 inhabitants) 2003–2012

Variables	Incidence rates of oral cancer (model 1)			Mortality rate of oral cancer (model 2)		
	β	CI95%	P	β	CI95%	P
FIXED EFFECT						
Contextual variables						
Per capita household income	–	–	–	–	–	–
% of older subjects	0.96	0.63: 1.30	<0.001	0.24	0.15: 0.33	<0.001
Proportion of adult smokers	0.29	0.07: 0.51	0.010			
Structure of PHC services						
FHST coverage	–	–	–	−0.01	−0.02: −0.003	0.006
Financing of PHC				−0.52^−9^	−0.96^−9^: −0.11^−9^	0.014
% Adequacy of SB equipment				–	–	–
Work process in PHC						
% of prevention and diagnosis of oral cancer	–	–	–	0.02	0.008: 0.04	0.002
RANDOM EFFECT						
Coefficient (β)	1.74			0.42		
Residue	0.65			0.39		

*β* regression coefficient, *CI 95%* 95% confidence interval, *P* Type I error probability (α)

(−-) not significant

## Discussion

### Main findings

Our findings highlighted the association of oral cancer mortality rates and the oral primary care. The exam of a time span data covering 10 years identified socioeconomic and demographic variables were predictors of oral cancer incidence rates. Variables related to the structure and work process in PHC were not associated with this outcome. However, indicators of socioeconomic and demographic context, structure, and working process in PHC were associated with oral cancer mortality rates.

It was also found that increased PHC funding and higher FHST coverage were associated with lower mortality rates of oral cancer. These results are unprecedented in both the national and international literature and demonstrate the importance of investing in PHC. A primary care model focusing in disease prevention and health promotion and based on interdisciplinary team, can provide a reduction in oral cancer mortality rates.

### Factors associated with the incidence rate of oral cancer

The proportion of elderly population presented significant positive association with oral cancer incidence rates. The mechanisms for suppressing the expression of oncogenes break down with aging [45–48], therefore aging is the main risk factor for cancer development [48]. The various stressors trigger cellular senescence, generating certain intracellular signals that modulate a distinct set of senescence-inducing signaling pathways leading to cancer [49, 50].

The proportion of smokers was higher in BFU with higher incidence rates of oral cancer. Although it is known that other factors besides smoking are required for initiation, promotion, and progression of cancer, several meta-analyses and systematic reviews have pointed smoking as a major risk factor for oral cancer [11, 51–53].

Other contextual variables such as gender ratio are not associated with the outcomes investigated. Historically, there was a higher incidence and mortality rates of oral cancer in men; however, this trend has shifted over the past few years [6, 54–56]. Thus, men and women should be target of policies towards coping with this important health problem.

Factors associated with mortality rates of oral cancer.

The proportion of elderly population also showed a significant, positive association with oral cancer mortality. It is known that elderly patients tend to experience more severe adverse effects of cancer treatments, particularly aggressive treatments, harming their quality of life and reducing survival rates [57, 58]. Because cancer is a potentially lethal disease [59], locations with high incidence rates also tend to have high mortality rates. This elderly population is not only at higher risk of development of the disease but also bears at greater risk of dying.

Populations with higher per capita household income had higher mortality rates of oral cancer. These results are similar to those of another ecological study conducted in Brazil [30], where locations with better social indicators had higher mortality rates of oral cancer. The authors found a correlation between increased life expectancy in locations with higher socioeconomic development and cancer mortality. Moreover, more developed centers, with better organization of health services, may have a better reporting system, which could increase the association between events. In order to assess the association between socioeconomic level and higher incidence of diagnosis of oral cancer, Johnson et al. [60] conducted a study using 2008 data from the American National Health Interview Survey (NHIS). The authors concluded that individuals of higher socioeconomic status were more likely to be diagnosed with oral cancer because they had more access to screening actions.

Many investigations have been conducted to assess the barriers to seeking treatment and the difficulties of professionals face for proper treatment of patients [23, 25, 61–64]. Low levels of knowledge on cancer, lack of financial resources, and fear of cancer diagnosis are some of the main obstacles for seeking health professionals [61–64]. An integrative literature review [24] discussed the reasons for which patients delay seeking professional help, identifying sociodemographic characteristics, health behaviors, and psychosocial factors. On the other hand, the omission of care by health teams has been associated with the absence of multidisciplinary work and insufficient attention to the needs of patients and community [23]. This creates a bottleneck effect and obstacle to providing comprehensive and resolute care for the patient. A study conducted in England pointed out that PHC general physicians are poorly prepared to suspect and diagnose malignant lesions in mouth and did not refer patients to OHT [65].

The Southeast and South regions of Brazil are the most developed and sites of referral centers for high complexity, including cancer diagnosis and treatment. There may be a migration of cases to such regions, a phenomenon already documented in the country by Naves et al. [66]. Therefore, although many studies indicate increased risk of development and death from oral cancer in people in areas of greater socioeconomic vulnerability [31, 55, 56, 60, 67], there is still uncertainty and limited knowledge about the relationship between socioeconomic factors and oral cancer. These studies were of individual basis and have shown inconclusive contradictory results [30, 67].

There is little data available on the costs of health services for treatment of patients with oral cancer in Brazil [68]. Using hospital admission data (AIH) paid for by SUS, in 2004 Pinto and Ugá [68] estimated that US$ 9,179,853.27 were spent on hospital admissions and US$ 14,450,238.87 were spent on chemotherapy for the treatment of lip, oral cavity and pharynx cancer. A study examining the cost-effectiveness of treating patients with head and neck cancer at an advanced stage found the average hospital cost per patient was US$ 2058.00 (chemoradiotherapy) and US$ 1167.00 (radiotherapy) in a SUS hospital. The incremental cost-effectiveness ratio was US$ 3300.00 per year. Increases in investment for prevention and early diagnosis actions would reduce health care costs and human suffering.

A BFU with a higher proportion of prevention actions and diagnosis of cancer also had higher mortality rates. Three hypotheses have been raised to explain these findings. First, more developed urban centers with better organization of the work process may have more services available, resulting in immigration of cases and increasing mortality rates recorded in these locations [66]. Secondly, it is possible that the oral health care model in Brazil is still not effectively identifying early stage cases. Finally, even if actions are offered, the health care network is not structured for timely service with appropriate referrals and case resolution.

One of the main guidelines of the National Oral Health Policy of 2004 was the expansion of the number of OHT in the family health strategy with a view to changing the care model in oral health [13, 14, 16, 17, 19, 69]. It also recommends conducting biopsy procedures by OHT in PHC or in Centers of Dental Specialties (CDS), with a focus to early diagnosis [15, 68, 69]. Until then, the model was essentially curative, individualized, performed by dentists in dental offices, focused on medication, and had large barriers to access due to restricted actions and services, especially for restorative and extraction treatment [16–19, 70]. Therefore, there was little potential to positively impact the oral health indicators of the Brazilian population [16, 17, 71].

Study limitations and strengths.

The study has limitations inherent to its design. The use of secondary data inserts potential selection biases due to the possibility of inadequate recording of events. However, national and international validated official databases were used. Moreover, the death cause registration is significantly improving in Brazil, increasing the validity of estimates for mortality rates [72, 73]. Additionally, data analysis at the BFU level does not take into account the impact of social inequality at the intra-state or intra-municipal levels, as well as the lower levels of aggregation. There are a small number of new cases and deaths due to oral cancer, so aggregation at a higher level is indicated. There are only 27 BFU, leading to a small sample size, therefore the adoption of a longitudinal design resulted in the expansion of the sample as each BFU was repeated several times. Despite this strength, caution is needed for inferences at an individual level because there is a risk of ecological fallacy.

The use of different data sources and the discontinuity of some indicators used hinder longitudinal comparisons. In addition, the hierarchical structure of longitudinal data, where repeated measurements are included within the BFU, generates dependence among observations made year by year and correlated errors. These assumptions require modeling of the data covariance matrix, which would not be achieved with conventional regression analyses. The linear regression of mixed effects adopted in this study produces estimates of standard errors of the model coefficients with lower defect as it incorporates the structure of data dependence in the estimates [43, 44, 74].

Finally, the use of population-based data and the standardization of mortality rates are two strengths of the study because they allowed the comparison of data at different times and among different locations. The pioneering nature of this study is also highlighted, which assesses the effect of socio-demographic indicators, the structure of oral health services, and the work process of PHC teams on the most recent incidence and mortality rates available for the country.

## Conclusion

Aspects of the structure and work process in primary healthcare in Brazil have effects on reducing oral cancer mortality, but not cancer incidence. Changes in the work process of oral health teams leading to more effective action in coping with oral cancer are needed. Investments in policies aimed at reducing risk factors should be made to improve the quality of care provided for the population, especially for the elderly, as well as increase the rate of early diagnosis by primary healthcare teams.

## Additional file

Additional file 1: Description of indicators (context, structure, process and outcome) and databases sources. Extension: .pdf. This file contain a full description of variables, as well as the data sources used to gather secondary information for the article. (PDF 209 kb)

## Abbreviations

ADHP: Advanced dental hygiene practitioner
BFU: Brazilian Federal Unit
BHU: Basic health units
DAB: Primary Care Department of the Ministry of Health
DHP: Dental hygiene practitioner
FACE/UFMG: Faculty of Administration and Economics of the Federal University of Minas Gerais
FHST: Family health strategy teams
HDI: Human Development Index
IBGE: Brazilian Institute of Geography and Statistics
ICD: International Code of Diseases
INCA: National Cancer Institute
IPE: Individual protection equipment
NHIS: American National Health Interview Survey
OHT: Oral Health Team
PHC: Primary health care
PMAQ-AB: National Program for Improving Access and Quality of Primary Care
PNSB: National Oral Health Policy
PNUD: United Nations Development Program
SAGE: System for Specialized Management Support
SD: Standard deviation
SIAB: Primary Care Information System
SIA-SUS: Ambulatory Information System;
SIM: Mortality Information System
VIGITEL: Surveillance of risk and protective factors for chronic diseases through telephone survey

## References

[1] Mogilner AR, Elishoov H (2015). Oral cancer--not only a disease of elder patients with risk factors. Refuat Hapeh Vehashinayim.

[2] Collaboration GB of DC. The global burden of cancer 2013. JAMA Oncol 2015;1(4):505–527. 10.1001/jamaoncol.2015.0735 PMC4500822 26181261

[3] Wünsch-Filho V, de Camargo EA (2001). The burden of mouth cancer in Latin America and the Caribbean: epidemiologic issues. Semin Oncol.

[4] Warnakulasuriya S (2009). Global epidemiology of oral and oropharyngeal cancer. Oral Oncol.

[5] INCA. Instituto Nacional de Câncer José Alencar Gomes da Silva. Câncer: tipos de câncer – boca. [documento on Internet]. 2016. [Cited 2016 may 12]. Avilable from: http://www2.inca.gov.br/wps/wcm/connect/tiposdecancer/site/home/boca/.

[6] Honorato J, Rebelo MS, Dias FL, Camisasca DR, Faria PA, Silva GA, Lourenço SQ (2015). Gender differences in prognostic factors for oral cancer. Int J Oral Maxillofac Surg.

[7] Zyl A Van, Attorney MJ. Aetiology of oral cancer. SADJ 2012;67(10):554–556. 23957094

[8] Khan Z, Tönnies J, Müller S. Smokeless tobacco and oral cancer in South Asia: a systematic review with meta-analysis. J Cancer Epidemiol. [document on Internet]. 2014. [Cited 2016 may 12]. Avilable from: http://www.hindawi.com/journals/jce/2014/394696/. 10.1155/2014/394696 PMC4109110 25097551

[9] Varoni EM, Lodi G, Iriti M (2015). Ethanol versus phytochemicals in wine: oral cancer risk in a light drinking perspective. Int J Mol Sci.

[10] Boing AF, Antunes JLF (2011). Socioeconomic conditions and head and neck cancer: a systematic literature review. Cien Saude Colet.

[11] Petti S, Masood M, Scully C. The magnitude of tobacco smoking-betel quid chewing-alcohol drinking interaction effect on oral cancer in South-East Asia. A meta-analysis of observational studies. PLoS One. 2013;8(11). [document on Internet]. [Cited 2016 May 10]. Avilable from: http://journals.plos.org/plosone/article/asset?id=10.1371%2Fjournal.pone.0078999.PDF 10.1371/journal.pone.0078999 PMC3832519 24260143

[12] Dantas TS, de Barros Silva PG, Sousa EF, da Cunha MP, de Aguiar AS, Costa FW, Mota MR, Alves AP, Sousa FB (2016). Influence of educational level, stage, and histological type on survival of oral cancer in a Brazilian population: a retrospective study of 10 years observation. Medicine (Baltimore).

[13] Almeida FC, Cazal C, Pucca Júnior GA, Silva DP, Frias AC, Araújo ME (2012). Reorganization of secondary and tertiary health care levels: impact on the outcomes of oral cancer screening in the São Paulo state. Brazil Braz Dent J.

[14] Torres-Pereira CC (2010). Oral cancer public policies: is there any evidence of impact?. Braz Oral Res.

[15] Torres-Pereira CC, Angelim-Dias A, Melo NS, Lemos CA, Oliveira EMF (2012). Strategies for management of oral cancer in primary and secondary healthcare services. Cad Saude Publica.

[16] Junqueira SR, Pannuti CM, de Mello Rode S (2008). Oral health in Brazil--part I: public oral health policies. Braz Oral Res..

[17] Chaves SCL, de Barros SG, Cruz DN, Figueiredo ACL, Moura BLA, Cangussu MCT (2010). Brazilian oral health policy: factors associated with comprehensiveness in health care. Rev Saúde Pública.

[18] Almeida GC, Ferreira MA (2008). Oral health in the context of the family health program: preventive practices targeting individual and public health. Cad Saúde Pública..

[19] Mattos GCM, Ferreira E, Leite ICG, Greco RM (2014). The inclusion of the oral health team in the Brazilian family health strategy: barriers, advances and challenges. Cienc Saude Colet.

[20] Souza FB, de Freitas e Silva MR, Fernandes CP, de Barro Silva PG, Alves APNN. Oral cancer from a health promotion perspective: experience of a diagnosis network in Ceará. Braz Oral Res. 2014;28(Spec No.):1–8. 10.1590/1807-3107BOR-2014.vol28.0018 24964281

[21] Macpherson LMD, McCann MF, Gibson J, Binnie VI, Stephen KW (2003). The role of primary healthcare professionals in oral cancer prevention and detection. Br Dent J.

[22] Brocklehurst P, Baker S, Speight P (2009). Factors affecting the referral of potentially malignant lesions from primary dental care: a pilot study in South Yorkshire. Prim Dent Care.

[23] Lombardo EM, da Cunha AR, Carrard VC, Bavaresco CS (2014). Delayed referrals of oral cancer patients: the perception of dental surgeons. Cien Saude Colet..

[24] Noonan B (2014). Understanding the reasons why patients delay seeking treatment for oral câncer symptoms from a primary health care professional: an integrative literature review. Eur J Oncol Nurs.

[25] Wade J, Smith HE, Hankins M, Llewellyn C (2010). Conducting oral examinations for cancer in general practice: what are the barriers?. Fam Pract.

[26] Brasil. Sala de Apoio à Gestão Especializada (SAGE). [document on Internet]. 2016. (Cited 2016 Feb. 15]. Available from: http://dab.saude.gov.br/portaldab/sala_apoio_gestao_estrategica.php

[27] Brasil. Departamento de Informática do SUS - DATASUS. Informações de Saúde (TABNET). [document on Internet]. 2016. (Cited 2016 Feb. 13]. Available from: http://www2.datasus.gov.br/DATASUS/index.php?area=02

[28] Dave B (2013). Why do GDPs fail to recognise oral cancer? The argument for an oral cancer checklist. Br Dent J.

[29] Hansen RP, Olesen F, Sørensen HT, Sokolowski I, Søndergaard J. Socioeconomic patient characteristics predict delay in cancer diagnosis: a Danish cohort study. BMC Heal Serv Res. 2008;8. [document on Internet]. 2016. (Cited 2015 Feb. 1]. Available from: http://www.ncbi.nlm.nih.gov/pmc/articles/PMC2311301/pdf/1472-6963-8-49.pdf 10.1186/1472-6963-8-49 PMC2311301 18307790

[30] Borges, de Lira DM, Sena, MF d, Ferreira, Fernandes MÂ (2009). Mortality for oral cancer and socioeconomic status in Brazil. Cad Saúde Pública.

[31] Conway DI, Brenner DR, McMahon AD, Macpherson LMD, Agudo A, Ahrens W (2015). Estimating and explaining the effect of education and income on head and neck cancer risk: INHANCE consortium pooled analysis of 31 case-control studies from 27 countries. Int J Cancer.

[32] Der Waal V, Are we able to reduce the mortality and morbidity of oral cancer (2013). Some considerations. Med Oral Patol Oral Cir Bucal.

[33] der Waal V, de Bree R, Brakenhoff R, Coebergh J-W (2011). Early diagnosis in primary oral cancer: is it possible?. Med Oral Patol Oral Cir Bucal.

[34] McGurk M, Chan C, Jones J, O'regan E, Sherriff M (2005). Delay in diagnosis and its effect on outcome in head and neck cancer. Br J Oral Maxillofac Surg.

[35] WHO (2005). Strengthening the prevention of oral cancer: the WHO perspective. 2005. Community Dent Oral Epidemiol.

[36] Mangalath U, Aslam SA, Khadar AHKA, Francis PG, Mikacha MSK, Kalathingal JH (2014). Recent trends in prevention of oral câncer. J Int Soc Prev Community Dent.

[37] Brasil. Programa Nacional de Melhoria do Acesso e da Qualidade da Atenção Básica (PMAQ) (2012). manual instrutivo.

[38] PNUD. Programa das Nações Unidas para o Desenvolvimento. Ranking IDHM Unidades da Federação 2010. [Document on Internet]. 2010. (Cited 2015 Feb. 1]. Available from: http://www.pnud.org.br/atlas/ranking/Ranking-IDHM-UF-2010.aspx.

[39] Brasil. Instituto Brasileiro de Geografia e Estatística. Censo [Document on Internet]. 2010. (Cited 2015 Feb. 1]. Available from: http://www.ibge.gov.br/home/

[40] Brasil. Vigilância de fatores de risco e proteção para doenças crônicas por inquérito telefônico. Vigitel [Document on Internet]. 2016. (Cited 2015 Feb. 1]. Available from: http://tabnet.datasus.gov.br/cgi/vigitel/vigteldescr.htm

[41] Avedis D (1992). The role of outcomes in quality assessment and assurance. QRB Qual Rev Bull.

[42] Avedis D (1976). Some basic issues in evaluating the quality of health care. ANA Publ.

[43] Rabe-Hesketh ASS (2008). Multilevel and longitudinal modeling using Stata.

[44] Tseng CH, Elashoff R, Li N, Li G (2016). Longitudinal data analysis with non-ignorable missing data. Stat Methods Med Res.

[45] Adams P, Jasper H, Rudolph L (2015). Aging-induced stem cell mutations as drivers for disease and cancer. Cell Stem Cell.

[46] Afanas I (2015). Mechanisms of superoxide signaling in epigenetic processes: relation to aging and cancer. Aging Dis.

[47] Lasry YB-NA (2015). Senescence-associated inflammatory responses: aging and cancer perspectives. Trends Immunol.

[48] Piano A, Titorenko V (2014). The intricate interplay between mechanisms underlying aging and cancer. Aging Dis..

[49] Judith C (2013). Aging, cellular senescence, and cancer. Annu Rev Physiol.

[50] Yossi ZYS. The ATM protein kinase: regulating the cellular response to genotoxic stress, and more. Nat Rev Mol Cell Biol. 2013;14:197–210. [Document on Internet]. (Cited 2015 Feb. 10]. Available from: http://www.nature.com/nrm/journal/v14/n4/execsumm/nrm3546.html

[51] Maleki D, Ghojazadeh M, Mahmoudi S-S, Mahmoudi S-M, Pournaghi-Azar F, Torab A (2015). Epidemiology of oral cancer in Iran: a systematic review. Asian Pac J Cancer Prev.

[52] Gupta NJB (2014). Systematic review and meta-analysis of association of smokeless tobacco and of betel quid without tobacco with incidence of oral cancer in South Asia and the Pacific. PLoS One.

[53] Weitkunat R, Sanders E, Lee PN (2007). Meta-analysis of the relation between European and American smokeless tobacco and oral cancer. BMC Public Health.

[54] Mimi C (2012). Women close the oral cancer gender gap. Todays FDA.

[55] Ferreira AJL, TOPORCOV TN, BIAZEVIC MGH, BOING AF, Bastos JL (2013). Gender and racial inequalities in trends of oral cancer mortality in Sao Paulo. Brazil Rev Saúde Pública.

[56] Auluck A, Walker BB, Hislop G, Lear NSSA, Rosin M (2014). Population-based incidence trends of oropharyngeal and oral cavity cancers by sex among the poorest and underprivileged populations. BMC Cancer.

[57] Kent E, Ambs A, Mitchell S, Clauser S, Smith AW, Hays R (2015). Health-related quality of life in older adult survivors of selected cancers: data from the SEER-MHOS linkage. Cancer.

[58] Van NA, Buffart L, Brug J, Leemans R, Leeuw IV (2015). The association between health related quality of life and survival in patients with head and neck cancer: a systematic review. Oral Oncol.

[59] Bobdey S, Balasubramanium G, Kumar A, Jain A (2015). Cancer screening: should cancer screening be essential component of primary health Care in Developing Countries?. Int J Prev Med.

[60] Johnson NW, Warnakulasuriya S, Gupta PC, Dimba E, Chindia M, Otoh EC (2011). Global oral health inequalities in incidence and outcomes for oral cancer: causes and solutions. Adv Dent Res.

[61] Patty G, Susan R, Stephen H, John I, William M, Brian OA (2011). Population-based study of factors associated with early versus late stage oral cavity cancer diagnoses. Oral Oncol.

[62] Awojobi S, Oluwatunmise S, Newton T (2012). Patients’ perceptions of oral cancer screening in dental practice: a cross-sectional study. BMC Oral Health.

[63] Shepperd J, Howell J, Logan HA (2014). Survey of barriers to screening for oral cancer among rural black Americans. Psychooncology.

[64] Jornet PL, Garcia G, Berdugo ML, Perez FP, Lopez AP-F (2015). Mouth self-examination in a population at risk of oral cancer. Aust Dent J.

[65] Mark G (2001). Primary care clinicians’ knowledge of oral cancer: a study of dentists and doctors in the north east of England. Br Dent J.

[66] Naves LA, Porto LB, Rosa JWC, Casulari LA, Rosa JWC (2015). Geographical information system (GIS) as a new tool to evaluate epidemiology based on spatial analysis and clinical outcomes in acromegaly. Pituitary.

[67] T-WHC-C W, Chang C-M, C-H Y, Wang Y-F, Lee C-C (2012). Effect of individual and neighborhood socioeconomic status on oral cancer survival. Oral Oncol.

[68] Pinto M, Ugá MAD (2010). The cost of tobacco-related diseases for Brazil's unified National Health System. Cad Saude Publica..

[69] Pedrazzi V, Dias KRHC, de Mello Rode S (2008). Oral health in Brazil - part II: dental specialty centers (CEOs). Braz Oral Res..

[70] De Souza A, Giusepp TMS (2007). Oral health in the Brazilian family health program: a health care model evaluation. Cad Saúde Pública..

[71] De Lucena PTC, Pucca Junior GA, Gomes EH (2010). Financing national policy on oral health in Brazil in the context of the unified health system. Braz Oral Res..

[72] De Sousa Queiroz R, Mattos IE, Monteiro GTR, Koifman S, Christine R (2003). Confiabilidade e validade das declaraçõeses de óbito por câncer de boca no Município do Rio de Janeiro. Cad Saúde Pública..

[73] Nogueira LT, CFN d R, KRO G, Campelo V (2009). Confiabilidade e validade das Declarações de Óbito por câncer de boca no Município de Teresina, Piauí, Brasil, no período de 2004 e 2005. Cad Saúde Pública..

[74] Fausto MA, Carneiro M, Antunes CM de F, Pinto JA, Colosimo EA (2008). Mixed linear regression model for longitudinal data: application to an unbalanced anthropometric dataset. Cad Saúde Pública..

