# Supplementary material for: Oral primary care: an analysis of its impact on the incidence and mortality rates of oral cancer
Source: BMC Cancer. 2017 Oct 30;17:706. doi: 10.1186/s12885-017-3700-z (PMC5661925; doi:10.1186/s12885-017-3700-z)
Supplement: Additional file 1: — Description of indicators (context, structure, process and outcome) and databases sources. Extension: .pdf. This file contain a full description of variables, as well as the data sources used to gather secondary information for the article. (PDF 209 kb) [file 12885_2017_3700_MOESM1_ESM.pdf]

Additional file 1. Description of indicators (context, structure, process and outcome) and databases sources

**Indicators**	**Source**	**Available years**	**Disaggregation level**
Socioeconomic and demographic			
GINI index	IBGE	2000 to 2012	Federative unit
Average per capita household income (sum of the monthly income of the household residents (Reals) divided by the number of inhabitants)	IBGE	2000 to 2012	
Proportion of elderly population (Number of people aged 60 or over divided by the total population)	IBGE	2002 to 2012	
Male/female ratio (Number of male inhabitants divided by the number of female inhabitants)	IBGE	2002 to 2012	
Proportion of adult smokers (Number of people aged 18 and over who currently smoke divided by the total number of adults)	VIGITEL	2007 to 2011	Federative unit capitals
BHU structure			
Average proportion of dental equipment (Number of minimum items in BHU, divided by the total BHU of the BFU - 8 items in 2002; 13 in 2008; 9 in 2012/13) – dental chair, seat, equipo, reflector, auxiliary unit, compressor and autoclave / stove	*1º PHC monitoring*	2002	*1ºPHC monitoring*<br>13,495 BHU/<br>13,973 FHST/<br>2,841 OHT<br><br>*2ºPHC monitoring*<br>1,875 BHU<br>2,133 FHST<br>1,858 OHT<br><br>*PMAQ-AB*<br>38,818 BHU<br>17,202 FHST<br>12,403 OHT
	2º PHC monitoring	*2008*	
	PMAQ-AB	*2012/13*	
Average proportion of dental clinical examination instruments (Number of minimum items in BHU, divided by the total BHU of the SU - 3 items: mirror, tweezers and explorer probe)	*1ºPHC monitoring*	2002	
	2ºPHC monitoring	*2008*	
	PMAQ-AB	*2012/13*	
Average proportion of individual protection equipment (IPE) inputs (Number of minimum items in BHU, divided by the total BHU - 5 items: gloves, masks, caps, glasses and apron)	*1º PHC monitoring*	2002	
	2º PHC monitoring	*2008*	
	PMAQ-AB	*2012/13*	
Proportion of full teams (Number of OHT composed of at least 1 dentist and 1 advanced dental hygiene practitioner (ADHP) or 1 dental hygiene practitioner (DHP)	*1º PHC monitoring*	2002	
	2º PHC monitoring	*2008*	
	PMAQ-AB	*2012/13*	
Proportion of dentist with no precarious bond work (Number of OHT where dentist has legally protected work link, divided by the total OHT)	*1º PHC monitoring*	2002	
	2º PHC monitoring	*2008*	
	PMAQ-AB	*2012/13*	

Indicators	Source	Available years	Disaggregation level
Financing of primary health care (PHC) (Annual Value (R$) of resources for PHC, divided by city population, multiplied by 1 million)	SAGE/ IBGE	2001 to 2012	Federative unit
Work process of FHST and OHT			
FHST coverage (Average number of FHST in the year, multiplied by 3,450 people, divided by the total municipal population)	SIAB/ IBGE	2002 to 2012	Municipalities
OHT coverage (Average number of OHT in the year, multiplied by 3,000 people, divided by the total municipal population)	SIAB/ IBGE	2002 to 2012	
Coverage of first dental appointment (Amount paid for first dental visit per year, divided by the population of that year, multiplied by 100)	SIAB/ IBGE	2000 to 2007	
Proportion of OHT performing preventive actions and oral cancer detection (No. of OHT performing prevention and oral cancer detection, divided by the total OHT)	*1º PHC monitoring*	2002	13,495 BHU<br>13,973 FHST<br>2,841 OHT
	2º PHC monitoring	*2008*	1,875 BHU<br>2,133 FHST<br>1,858 OHT
	PMAQ-AB	*2012/13*	38,818 BHU<br>17,202 FHST<br>12,403 OHT
Average individual basic dental procedures (Annual quantity of dental procedures, divided by the population of that year)	SIA-SUS/ IBGE	2000 to 2006	Federative unit
Supervised tooth brushing (Number of people participating in collective actions of supervised tooth brushing held in a certain place and period divided by the population)	SIA-SUS/ IBGE	2012/13	
Results			
Estimated incidence rate of oral cancer (Number of estimated cases of oral cancer (ICD10: C00-C10) in the reference year, divided by the total local population in the same year multiplied by 100,000)	INCA/ IBGE	2003, 2008 and 2012	Federative unit
Mortality rate for oral cancer (No. of registered deaths from mouth cancer (ICD10: C00-C10) in the reference year, divided by the total local population in the same year multiplied by 100,000)	SIM/ IBGE	2000 to 2012	

IBGE: Brazilian Institute of Geography and Statistics. VIGITEL: Surveillance of risk and protective factors for chronic diseases through telephone survey. BHU: Basic Health Unit. FHST: Family Health Strategy Team. OHT: Oral Health Team. BFU: Brazilian Federal Unit. PHC: Primary Health Care. SIA-SUS: Ambulatory Information System. INCA: National Cancer Institute. ICD: International Code of Diseases. SIM: Mortality Information System. IPE: individual protection equipment. SIAB: Primary Care Information System. SAGE: System for Specialized Management Support. PMAQ-AB: National Program For Improving Access and Quality on Public Primary Health Care. ADHP: Advanced Dental Hygiene Practitioner. DHP: Dental Hygiene Practitioner.
